# Microscopic agglutination test on captive rattlesnakes : Data on serovars and titers

**DOI:** 10.1016/j.dib.2016.03.050

**Published:** 2016-03-18

**Authors:** T.C.S. Rodrigues, A.L.Q. Santos, A.M.C. Lima, D.O. Gomes, G.F. Cardoso, V.L.C. Brites

**Affiliations:** a Wildlife Teaching and Research Laboratory (LAPAS), College of Veterinary Medicine (FAMEV), Federal University of Uberlândia (UFU), Avenida Amazonas 2245, Jardim Umuarama, 38405-302 Uberlândia, MG, Brazil; b Laboratory of Infectious Diseases, College of Veterinary Medicine (FAMEV), Federal University of Uberlândia (UFU), Rua Ceará, s/n, Bloco 2D, Sala 33, Campus Umuarama, 38405-315 Uberlândia, MG, Brazil; c Reptile Sector, Institute of Biology, Federal University of Uberlândia (UFU), Rua Ceará, s/n, Bloco 2D, Campus Umuarama, 38405-315 Uberlândia, MG, Brazil

## Abstract

The microscopic agglutination test (MAT) is considered the “golden standard” leptospirosis serodiagnostic test, but there is little information about it as it pertains to snakes. To fill this information gap, we provide data on serovars and titers of fifty-six *Crotalus durissus collilineatus* sera samples that tested positive by MAT (10.1016/j.actatropica.2016.02.006 (Rodrigues et al., 2016) [5]). These data are presented in a table, along with a description of the methodology used for sample collection and serologic testing.

**Specifications Table**

**Table:** 

Subject area	Biology
More specific subject area	Reptile leptospirosis
Type of data	Table
How data was acquired	Baby 2-Fanem Centrifuge; Axio Scope.A1 Microscope, Zeiss
Data format	Raw
Experimental factors	Blood was collected from the rattlesnakes and centriguged for serum analysis
Experimental features	Data on serovars and titers of serologic positive *C. durissus collilineatus* were obteined using microscopic agglutination test (MAT)
Data source location	Uberlândia, Minas Gerais State, Brazil
Data accessibility	Data are with this article

**Value of the data**

• These data can be compared with MAT results from reptile breeding centers and zoos around the world, aiding in the development of future comparative studies.
• These data provide a basis for future epidemiologic studies and may facilitate the study design of future researches on the theme.
• These data may encourage further research on rattlesnake zoonotic diseases.

## 1 Data

We make available data on leptospirosis serodiagnosis by the microscopic agglutination test on fifty-six positive Crotalus durissus collilineatus rattlesnakes. Data are presented in Table 1, showing seroreactivity to twenty-two different serovars, with titers varying from 25 to 1600 [5]. Each animal listed (under a specific identification sequence) was positive to at least one serovar. Animal identification, sex and positive serovars with respective titers are also provided.

## 2 Experimental design, materials and methods

Data was obtained with the authorization of the Brazilian Institute of Environment and Renewable Natural Resources – IBAMA (SISBIO Permit No. 46845) and the approval of the Ethics Committee on Animal Use – CEUA/UFU (Protocol 120/14).

### 2.1 Subjects

Sera samples of fifty-six clinically healthy adult *C. durissus collilineatus* were utilized. The animals belonged to the Reptile Sector (Conservation Breeding Center for Scientific Purposes) of the Federal University of Uberlândia (UFU) and were sent to the breeding center by environmental authorities or by members of the community, because they were found in rural, peri-urban and urban areas of the cities of Uberlândia and Araguari, Minas Gerais state.

### 2.2 Sample collection

The rattlesnakes were restrained by trained handlers using previously described techniques [1]. Blood was drawn by puncture of the vertebral venous plexus [2] after disinfection with 70% alcohol, using 13×4.5 mm disposable hypodermic needles and 3 ml disposable syringes.

The blood samples were transferred to tubes without anticoagulant and centrifuged at 720 g for 10 minutes using a Baby 2-Fanem centrifuge. Serum samples were placed in individually identified microtubes and then stored at −20 °C until the MAT was performed.

### 2.3 Microscopic Agglutination Test (MAT)

The MAT was performed at the Laboratory of Infectious Diseases of UFU and a cutoff dilution of 1:25 was established, which was previously used on crocodilians [3]. A panel of 22 serovars was used: Andamana, Autumnalis, Australis, Bataviae, Bratislava, Canicola, Cynopteri, Copenhageni, Djasiman, Grippotyphosa, Hardjo, Hebdomadis, Icterohaemorrhagiae, Javanica, Panama, Patoc, Pomona, Pyrogenes, Sentot, Tarassovi, Whitcombi and Wolfii.

On flat bottom microwell plates it were placed 23 μL of saline 0.9%, 2 μL of serum from each animal, and 25 μL of antigen (each of the 22 serovars) in each well, resulting in a final solution of 50 µL. This final solution was gently stirred by hand and allowed to rest for one hour at room temperature. Reading was then carried out by dark field microscopy directly from the plate wells, using a 10X objective lens and eyepiece (Axio Scope.A1 Microscope, Zeiss). Samples showing agglutination of more than 50% of the field were considered positive [4].

The samples considered positive were then subjected to antibody titration. This involved subjecting the serum of each sample to consecutive two-fold dilutions (1:50, 1:100, 1:200, 1:400, 1:800, 1:1600 and 1:3200), including the antigen. Reading was performed after the same resting time used at room temperature and in the same microscope used initially. The titer of each sample was the highest dilution at which agglutination corresponded to 50% or more of the field. Samples can be considered positive to more than one serovar, due to cross-reactions or exposure to multiple serovars [4].

## Figures and Tables

**Table 1:** Animal identification, sex and seroreactive serovars with respective titers of fifty-six *Crotalus durissus collilineatus* positive to the microscopic agglutination test.

**Animal Identification**	**Sex**	**Serovar (Titer)**
V A	F	Panama (50), Whitcombi (100)
T B	F	Whitcombi (50)
F A	F	Panama (50), Patoc (50)
28 B	M	Whitcombi (1600), Sentot (200), Patoc (200), Panama (1600), Javanica (400), Djasiman (400), Wolfii (200), Tarassovi (400)
80 B	M	Bataviae (50), Bratislava (50), Canicola (50), Copenhageni (50), Hebdomadis (50), Pyrogenes (50), Whitcombi (50)
V B	F	Hebdomadis (50)
H B	F	Hebdomadis (50), Javanica (100)
40 maior	F	Patoc (50)
61 B	M	Autumnalis (50), Copenhageni (50), Canicola (50), Bratislava (50), Australis (50), Grippotyphosa (50), Hebdomadis (100), Pyrogenes (50), Tarassovi (50), Wolfii (50), Javanica (100), Patoc (100), Whitcombi (100)
B A	F	Javanica (50)
X B	F	Bataviae (50), Javanica (50)
61 A	F	Patoc (100), Javanica (50)
3 B maior	F	Javanica (100), Patoc (50)
4 A	F	Djasiman (50), Andamana (50), Cynopteri (50), Panama (50), Patoc (50), Whitcombi (50)
5 A	M	Andamana (50), Javanica (50), Whitcombi (50)
5 B	M	Andamana (50), Whitcombi (50), Patoc (50), Panama (50), Javanica (50)
U B	M	Andamana (100), Javanica (100), Whitcombi (50)
X A	M	Whitcombi (50), Patoc (50), Javanica (50)
52 A	M	Patoc (50), Javanica (25), Andamana (50)
O A	F	Andamana (50), Javanica (25)
P A	F	Andamana (50), Javanica (25)
E A	F	Andamana (50), Javanica (25), Patoc (50)
8 A menor	M	Andamana (50), Javanica (25)
D B	M	Pyrogenes (25), Andamana (50), Javanica (25)
62 B maior	M	Andamana (50), Javanica (25), Whitcombi (25), Patoc (25)
O B	F	Andamana (50), Javanica (25), Panama (400)
E B	F	Andamana (50), Javanica (25), Patoc (50)
D A	M	Andamana (50), Javanica (50), Patoc (25), Whitcombi (25)
S B	M	Andamana (50), Javanica (50), Patoc (25)
H A	F	Andamana (50), Javanica (50), Patoc (25)
M menor	F	Andamana (50), Javanica (25), Patoc (25)
L A	M	Autumnalis (50), Australis (50), Bataviae (25), Bratislava (25), Canicola (25), Copenhageni (25), Grippotyphosa (25), Hardjo (25), Hebdomadis (25), Icterohaemorrhagiae (25), Pomona (25), Pyrogenes (50), Tarassovi (25), Wolfii (25), Djasiman (25), Andamana (50), Cynopteri (25), Javanica (25), Panama (50), Patoc (50), Whitcombi (25)
S A	M	Andamana (50), Javanica (25), Patoc (25), Panama (50)
4 B	F	Pomona (50), Pyrogenes (50), Patoc (25), Javanica (25), Andamana (50)
Y A	M	Javanica (25), Andamana (50)
76 maior	M	Javanica (25), Andamana (50)
52 F	F	Javanica (25), Andamana (50)
J A	F	Javanica (25), Andamana (50), Patoc (800)
6 B maior	M	Javanica (50), Andamana (50), Patoc (50)
7 A maior	F	Javanica (50), Andamana (50), Patoc (25)
102 maior	F	Javanica (25), Andamana (50), Patoc (25), Whitcombi (25)
79 B	F	Javanica (100), Andamana (50)
Y B	F	Icterohaemorrhagiae (100), Bratislava (25), Djasiman (100), Javanica (25), Panama (200)
79 A	M	Javanica (25), Andamana (50)
3 menor	F	Andamana (50), Javanica (25), Patoc (25)
R A	F	Whitcombi (25), Javanica (50), Andamana (50)
C B	F	Javanica (25), Patoc (25)
50 B	M	Javanica (50), Andamana (50)
91 A menor	M	Andamana (50), Javanica (25)
9 A menor	M	Panama (50), Javanica (50)
52 C	M	Javanica (25), Patoc (25)
28 A	M	Whitcombi (25), Patoc (25), Panama (25), Javanica (25)
42	M	Javanica (25)
P B	F	Bataviae (25), Tarassovi (25)
10 A menor	M	Patoc (25), Panama (25), Javanica (25)
K A	M	Pyrogenes (50), Pomona (50), Autumnalis (25), Australis (25), Bataviae (25), Bratislava (25), Canicola (25), Copenhageni (25), Grippotyphosa (25), Hardjo (25), Hebdomadis (25), Icterohaemorrhagiae (25), Tarassovi (25), Wolfii (25), Djasiman (25), Andamana (25), Cynopteri (25), Javanica (25), Panama (100), Patoc (25), Whitcombi (50)
