# Supplementary material for: Microscopic agglutination test on captive rattlesnakes : Data on serovars and titers
Source: Data Brief. 2016 Mar 18;7:877–81. doi: 10.1016/j.dib.2016.03.050 (PMC4816860; doi:10.1016/j.dib.2016.03.050)
Supplement: Supplementary file 1 — Supplementary material [file mmc1.docx]

The authors attests that there are no conflicts of interest.
